# Unintended medication discrepancies and associated factors upon patient admission to the internal medicine wards: identified through medication reconciliation

**DOI:** 10.1186/s12913-022-08628-5

**Published:** 2022-10-15

**Authors:** Tilaye Arega Moges, Temesgen Yihunie Akalu, Faisel Dula Sema

**Affiliations:** 1 Debre Tabor University, Debre Tabor, Ethiopia; 2 Department of Epidemiology and Biostatistics, Institute of Public Health, College of Medicine and Health Sciences, University of Gondar, Gondar, Ethiopia; 3 Department of Clinical Pharmacy, School of Pharmacy, College of Medicine and Health Sciences, University of Gondar, Gondar, Ethiopia

**Keywords:** Medication discrepancy, Medication reconciliation, Hospital admission, Pharmacist’s intervention, Medication error

## Abstract

**Background:**

Medication reconciliation (MedRec) is a widely accepted tool for the identification and resolution of unintended medication discrepancies (UMD).

**Objective:**

This study aimed at assessing the magnitude and associated factors of UMD identified through medication reconciliation upon patient admission to the internal medicine wards.

**Methods:**

Prospective cross-sectional study was conducted at the internal medicine wards of Felege Hiwot and Tibebe Ghion comprehensive specialized hospitals in Bahir Dar city, Northwest Ethiopia, from May 01 to July 30, 2021. Data were collected by using a data abstraction format prepared based on standard MedRec tools and previous studies on medication discrepancy. Pharmacists-led MedRec was made by following the WHO High5s “retroactive medication reconciliation model”. SPSS® (IBM Corporation) version 25.0 was used to analyze the data with descriptive and inferential statistics. A binary logistic regression analysis was used to identify factors associated with UMD. A statistical significance was declared at a *p*-value < 0.05.

**Results:**

Among 635 adult patients, 248 (39.1%) of them had at least one UMD. The most frequent types of UMDs were omission (41.75%) and wrong dose (21.9%). The majority (75.3%) of pharmacists’ interventions were accepted. Polypharmacy at admission (*p*-value < 0.001), age ≥ 65 (*p*-value = 0.001), a unit increase on the number of comorbidities (*p*-value = 0.008) and information sources used for MedRec (*p*-value < 0.001), and medium (*p*-value = 0.019) and low adherence (*p*-value < 0.001) were significantly associated with UMD.

**Conclusion:**

The magnitude of UMD upon patient admission to the internal medicine wards was considerably high. Omission and the wrong dose of medication were common. Older age, polypharmacy, low and medium adherence, and an increase in the number of comorbidities and information sources used for MedRec are significantly associated with UMDs. Pharmacists' interventions were mostly acceptable. Thus, the implementation of pharmacists-led MedRec in the two hospitals is indispensable for patient safety.

**Supplementary Information:**

The online version contains supplementary material available at 10.1186/s12913-022-08628-5.

## Background

Patient safety is one ofthe most important components of health care delivery, which is essential for achieving universal health coverage and moving towards the UN sustainable development goals. However, a medication error is inflicting significant patient harm and financial burden on hospitalized patients [1, 2]. The annual cost associated with it has been estimated to be $42 billion, or nearly 1% of the total global health expenditure [3].

In 2017, the world health organization (WHO) launched its third global safety challenge, called “Medication without Harm,” aimed at reducing serious and preventable medication-related harm worldwide by 50% [3, 4]. One of the strategies established to reduce medication errors is the practice of medication reconciliation (MedRec) [5, 6]. It is a formal process that involves matching the medicines that the patient should be prescribed with those that are actually prescribed, and it involves adequately reporting any therapy change [7]. Its main objective is to reduce a possible unintended medication discrepancy (UMD), which is one of the major causes of medication errors and related adverse drug events (ADEs) at the transition of care [8–12]. UDM occurs if the prescriber unintentionally changed, added, or omitted a medication that the patient had been taking before the transition of care [13].

UMD is an important public health problem globally [4, 8, 12]. More than 60% of medication errors related to UMD occur during the transition of care, as the admission process is one of the most vulnerable areas in terms of medication safety [4, 8]. Literature indicated that about 3–97% of adult patients had at least one UMD at admission [14]. Omissions (35.49–98.3%) and wrong dose (7- 40.3%) are the most frequent types of UMDs [12, 15–31].

Medication error resulting from UMDs is among the primary causes of morbidity and mortality among hospitalized patients [32]. It results in considerable patient harm with substantial clinical and economic consequences [7, 33, 34]. It can also lead to ineffective pharmacotherapy, interruptions of treatment, adverse drug events, longer hospital stays, and an increase in hospital readmission, which all increase health care costs [13, 20, 34].

Taking multiple medications at home, having polypharmacy upon admission to a hospital, older age, being female, and frequent admission to a hospital in the past one year are among the frequently reported factors of UMD [18, 27, 35–38].

A variety of existing evidence has demonstrated that a structured MedRec that involves the best possible medication history (BPMH), which reflects an accurate and complete list of all medications taken prior to admission, is an effective technique for preventing, identifying, and rectifying numerous clinically relevant UMDs and associated ADEs by improving the quality of communication at the transition of care [12, 13, 22, 33, 39–42]. Moreover, pharmacists’ led-MedRec has shown a positive impact on improving patients’ care [27, 41, 43].

Most of the studies that quantified medication discrepancies were undertaken in high and middle-income countries [4, 15–27, 38, 44–46]. However, to the best of the authors' literature review, there is only limited evidence in Africa [47, 48]. Moreover, the previous studies done in eastern and central Ethiopia have not included patients at admission, and they have not reported the pharmacists’ intervention and the rate of acceptance of their recommendations to rectify the identified UMDs [28, 49]. So, the aim of the current study was to assess the magnitude and associated factors of UMDs identified through medication reconciliation during patient admission to the internal medicine wards of Felege Hiwot and Tibebe Ghion comprehensive specialized hospitals in Bahir Dar city, Northwest Ethiopia. It may help to promote the implementation of a formal MedRec process at a transition of care and the integration of pharmacists in a health care team to improve patient safety.

## Methods

### Study period and area

This cross-sectional study was conducted at Felege Hiwot comprehensive specialized hospital (FHCSH) and Tibebe Ghion comprehensive specialized hospital (TGCSH) in Bahir Dar city, Northwest Ethiopia from May 01 to July 30, 2021. Bahir Dar is the capital city of Amhara national regional state located 554 km far from Addis Ababa, Ethiopia in the Northwest direction. These hospitals are the only governmental comprehensive specialized hospitals in the city serving more than 5.5 million people in the catchment area. They are also serving as teaching hospitals for Bahir Dar University. FHCSH has 800 beds for inpatient services. Of these, 74 are for internal medicine wards. TGCSH is a newly established hospital in 2018. The hospital has 500 beds for inpatient service. Of these, 79 beds are under the department of internal medicine.

### Population, inclusion, and exclusion criteria

Adult patients admitted to the internal medicine wards of FHCSH and TGCSH were the source population. However, adult patients admitted to the internal medicine wards of FHCSH and TGCSH from May 01 to July 30, 2021, were the study population. Patients having at least one medication for chronic use before admission were included. Patients were excluded from the study if they were not willing to participate in the study and too ill to respond to the interview questions and/or did not have a caregiver.

### Sample size determination and sampling procedure

The sample size was determined by using a single population proportion formula with the assumption of a 95% confidence level, 5% margin of error, and 50% proportion. Since the prevalence of UMD at admission to the internal medicine ward is not known in Ethiopia, a 50% proportion was used.

$n=\frac{{z}^{2}\left(p\right)\left(1-p\right)}{{d}^{2}}$ = $\frac{{\mathrm{1,96}}^{2}\left(0.50\right)\left(1-0.50\right)}{({0.05)}^{2}}$  = 384; by using contingency of 10% i.e. 384*10% = 39; the calculated sample size was 384 + 39 = 423. Intentionally adding 50% to this sample to increase the power of the study, the final sample size was 423*50% = 635. Where, d = margin of error, *p* = proportion of sample population, Za/_2_ = the value under standard normal table using a 95% confidence interval, and *n* = the sample size.

**Fig. 1:**
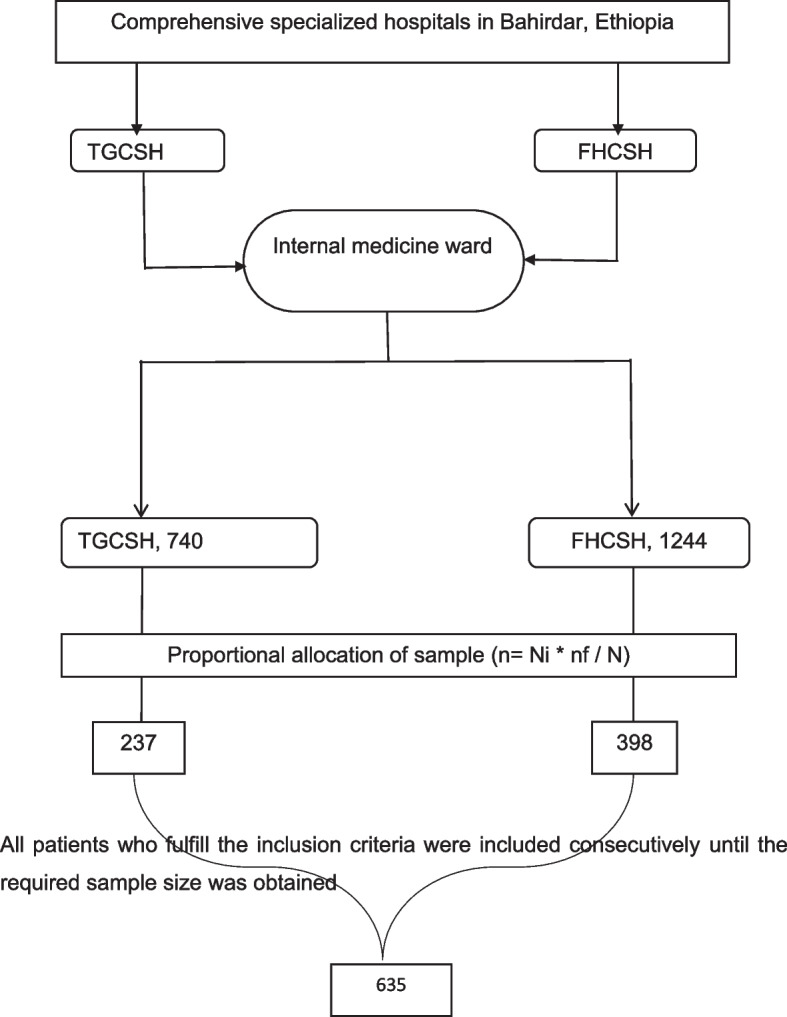
Diagrammatic representation of the sampling procedure. n: the total sample size selected from the internal medicine wards of the two hospitals; N: the total population from the internal medicine wards of both hospitals; Ni: the total population from the internal medicine wards of each hospital; nf: the final sample size; FHCSH: Felege Hiwot comprehensive specialized hospital; TGCSH: Tibebe Ghion comprehensive specialized hospital

According to the 2019/2020 annual report of the hospitals, 4,975 and 2,976 patients were admitted to the internal medicine wards of FHCSH and TGCSH, respectively. Considering the data collection period of 3 months, the total sample size was allocated proportionally to each hospital as follows (Fig. 1): n $=\frac{{\varvec{n}}{\varvec{f}}\boldsymbol{*}{\varvec{N}}{\varvec{i}}}{{\varvec{N}}}$. Where, *n* = the total sample size selected from the internal medicine wards of the two hospitals; *N* = the total population from the internal medicine wards of both hospitals; Ni = the total population from the internal medicine wards of each hospital, and nf = the final sample size (635). FHCSH: n1 = 635*1244/1984 = 398 patients; TGCSH: n2 = 635*740/1984 = 237 patients. Finally, patients who fulfilled the inclusion criteria were selected based on their random arrival to the internal medicine wards of the hospitals consecutively until the required sample is obtained in both hospitals.

### Variables of the study

The dependent variable was the presence of at least one UMD upon admission to the internal medicine wards of the hospitals. The independent variables include the socio-demographic characteristic of the participants (e.g. age), clinical characteristics (e.g. type of admission, number of admissions in the last 12 months, and number of comorbidities), and medication-related factors (medication adherence, source of medication fee, number of prescribed medications at admission, number of previous or home medications, and number of information sources consulted for the BPMH).

### Operational definitions

Medication discrepancy is any difference between the BPMH and the admission medication orders including prescription medications and non-prescription medications like over-the-counter (OTC) medicines, vitamins, and minerals [50].

Intended medication discrepancies are clinically understandable and appropriate discrepancies between the BPMH and the admission orders based on the patient’s plan of care [8, 50].

Unintended medication discrepancy is one in which the prescriber unintentionally changed, added, or omitted a medication the patient was taking before admission [8, 13, 50]; it includes, but is not limited to the following:

A. Omission: a deletion of a pre-admission medication with no supporting clinical explanation or documentation for the omission.
B. commission: Incorrect addition of medication not part of the patient’s pre-admission medication and there is no clinical explanation or documentation for adding the medication to the patient’s therapy.
C. wrong dose, route, and frequency: different dosage, route, or frequency of medication than what the patient reports taking before hospitalization is ordered at admission. The differences are not explained by changes in the patient’s clinical status at admission such as renal or hepatic function.
D. duplication: when two or more medications of the same group are prescribed without a plausible explanation considering a medication used before admission.
E. drug interaction: when two or more medications are not in the same group, but are unnecessary to be used concomitantly and interact with each other considering a medication used before admission.
F. Wrong duration: when the duration of a medication’s regimen is different from current evidence-based treatment guidelines considering a medication used before admission.

MedRec: Identifying discrepancies between the BPMH and any currently prescribed medications at admission [51].

BPMH: a medication history obtained by a clinician who includes a thorough history of all regular medication use (prescribed and non-prescribed), using several different sources of information (patient interview, family member, medical record, home medications bottles or boxes, and any other sources obtained) [51]. In this study, pharmacists obtained medication history was taken as a gold standard.

Polypharmacy: The concurrent use of multiple medications i.e. routine use of five and above medications [52].

The acceptance of intervention is defined as a modification in the medication therapy planning as suggested by the pharmacist to the prescribing physician.

### Data collection tool, procedure, and quality control

Two pharmacists who were trained on the study’s purpose, methods, and the medication reconciliation process collected the data prospectively by using a data collection tool prepared based on standard tools and published validation studies [8, 15, 16, 18, 28, 49, 50, 53]. To check the face validity of the data collection tool, it was sent to two faculty members and one physician. The data collection tool was pretested on 5% of (32) patients at the university of Gondar comprehensive specialized hospital. The data collected for the pretest was not included in the final analysis. Within 24–48 h of patient admission, the pharmacists’ obtained the BPMH from at least two sources of information before communicating with the responsible physician. The MedRec was made by following the WHO High5s “retroactive medication reconciliation model” as follows: a medication history which leads to admission medication orders were made by the treating physicians. A BPMH was done by the pharmacists within 24–48 h of the decision made for patient admission, concurrently. Then, the BPMH was compared with the admission medication orders. When discrepancies were found, the most responsible treating physician was communicated to differentiate between the undocumented intentional and unintentional discrepancies. To rectify the UMD, the pharmacists made a discussion with the treating physician, the patient, and their attendants based on the nature of the discrepancy. The acceptance of the recommendations made by the pharmacists was checked within 24–48 h after the discussion. Considering WHO high 5 s Standard Operating Protocol (SOP), the name, dose, frequency, route, and duration of treatment were collected for each prescribed, and non-prescribed medication. Medications involved in UMDs were categorized by using the WHO Anatomical Therapeutic Chemical classification (ATC) for medication class [54], and UMDs were grouped by using the medication discrepancy taxonomy (Med Tax) [55]. The proximal cause of the medication discrepancy was classified per the Institute of Safe Medication Practices (ISMP) [56]. Whereas, both the pharmaceutical interventions and outcomes (acceptance or non-acceptance) were classified according to the Pharmaceutical Care Network Europe (PCNE) 2019 [57]. The classification of home medication groups was based on the 2020 Ethiopian essential medicine list [58].

Medication adherence was assessed using the Adherence in Chronic Diseases Scale (ACDS) because all patients were using at least one medication for a chronic condition. The tool is available free of charge on the website of the Department of Health Promotion, Collegium Medicum, Nicolaus Copernicus University, Poland [59]. The questions used for assessing medication adherence were translated to the Amharic language by experts in the area, and back-translated to the English language before the pretest to minimize translation errors. Alpha Cronbach test was used to measure the internal consistency of the medication adherence tool, and an index of 0.82 was obtained. The tool consists of seven questions. The first 5 questions addressed the patient's behavior related to the medication regimen and the last 2 were dealing with the patient-physician relationship. The answer for each of the questions was rated from 0 to 4 points. Which makes the sum of the total medication adherence score range from 0 to 28. Medication adherence was labeled as a high, medium, and low if the total score was > 26 points, 21–26 points, and < 21 points respectively. The data collection was supervised and data were checked for consistency, and completeness on daily basis.

### Data analysis

The data were entered into the EpiData software version 4.6.0.0, and then exported to Statistical Package for the Social Sciences (SPSS®) version 25.0 for analysis. Descriptive statistics such as mean and standard deviations for normally distributed continuous data, median and interquartile range (IQR) for non-normally distributed continuous data, and frequencies and percentages for categorical data were done. A binary logistic regression analysis was carried out to identify factors associated with UMD. All independent variables with *p* < 0.2 in the univariate analysis were used for the multi-variable analysis using the enter method [60]. Variance inflation factor (VIF) and Shapiro–Wilk test were used to check the multicollinearity and normality of the data, respectively. All included variables were with a variance inflation factor (VIF) < 5 [61, 62]. The data were considered normally distributed if the Shapiro–Wilk test was with a *p*-value > 0.05. Hosmer and Lemeshow test was used for checking the model goodness of fit. A *p*-value of < 0.05 was used to declare the statistical significance.

## Results

### Socio-demographic characteristics of the study participants

From a total of 635 patients, more than half (53.7%) were males (95% confidence interval (CI) = 49.7, 57.6). The majority (55.2) (95% CI = 53.7, 61.5) of the participant were residents of rural area, and 42.5% (95% CI = 38.6, 46.5) were farmers. The median (IQR) age of the patients was 60 (49 to 69) years. More than three fourth of them (67.6%) (95% CI = 63.8, 71.2) were married. About one-third (35%) (95% CI = 31.3, 38.8) of patients completed only primary education (Table 1).

**Table 1:** The socio-demographic characteristics of the study participants

Variable	Categories	Hospital		
		FHCSH; N (%)	TGCSH; N (%)	Total N (%)
Sex	Male	220 (55.3)	121 (51.1)	341 (53.7)
	Female	178 (44.7)	116 (48.9)	294 (46.3)
Age (years)	< 65	230 (58)	167 (70.5)	397 (62.5)
	≥ 65	168 (42.2)	70 (29.5)	238 (37.5)
Residence	Urban	196 (73)	73 (27)	269 (42.4)
	Rural	202 (55.2)	164 (44.8)	366 (57.6)
Marital status	Single	115 (28.9)	41 (17.3)	156 (24.6)
	Married	250 (62.8)	179 (75.5)	429 (67.6)
	Divorced	25 (6.3)	13 (5.5)	38 (5.98)
	Widowed	8 (2)	4 (1.7)	12 (1.9)
Educational level	Cannot read and write	37 (9.3)	10 (4.2)	47 (7.4)
	Non-formal education	31 (7.8)	35 (14.8)	66 (10.4)
	Primary education (1–8 grade)	128 (32.2)	94 (39.7)	222 (35)
	Secondary education (9–12 grade)	103 (25.9)	52 (21.9)	155 (24.4)
	Tertiary education (diploma and above)	99 (24.9)	46 (19.4)	145 (22.8)
Job-status	Housewife	24 (6)	20 (8.4)	44 (6.9)
	Farmer	159 (39.9)	111 (46.8)	270 (42.5)
	Unemployed	48 (12)	28 (11.8)	76 (12)
	Civil servant	55 (72.4)	21 (8.9)	76 (12)
	Merchant	75 (13.8)	30 (12.7)	105 (16.5)
	Other	37 (9.3)	27 (11.4)	64 (10.1)

*FHCSH* Felege Hiwot comprehensive specialized hospital, *TGCSH* Tibebe Ghion comprehensive specialized hospital, *N* Frequency, % Percent

### Clinical and medication-related characteristics of the study participants

More than half (54.8%) of patients had at least two comorbidities. Cardiovascular diseases were the most frequent comorbidities (31.6%) and reason for admission (32.9%). The out-of-pocket payment was the source of medication fees for around two-thirds (64.9%) of patients, and more than half of patients (57.6%) managed their preadmission medications autonomously. Medication adherence for the majority (62.4%) (95% CI = 58.5, 66.1) of study participants was low. The median (IQR) number of home medications per patient was 2 (1 to 2), with a total number of 1285 medications. The median (IQR) number of medications upon hospital admission was 5 (3 to 6) per patient with a total number of 3,017 medications. Cardiovascular drugs were the most frequent (28.9%) home medications followed by anti-infective (25.7%) and respiratory medicines (15.5%) (Table 2).

**Table 2:** Clinical and medication-related characteristics of the study participants

Variable	Categories	N (%)
Comorbidities by disease classification	Respiratory diseases	203 (18.1)
	Infectious disease	239 (21.3)
	Gastrointestinal diseases	117 (10.4)
	Cardiovascular diseases	370 (32.9)
	Malnutrition diseases	10 (0.9)
	Obstetrics and gynecological diseases	5 (0.4)
	Hematological malignancies	15 (1.3)
	Musculoskeletal and joint diseases	58 (5.2)
	Endocrine disorder diseases	106 (9.4)
	Total^a^	1123 (100)
Reasons for admission	Respiratory diseases	240 (19.8)
	Infectious diseases	254 (20.9)
	Gastrointestinal diseases	115 (9.5)
	Cardiovascular diseases	384 (31.6)
	Malnutrition diseases	18 (1.5)
	Hematological malignancies	6 (0.5)
	Musculoskeletal and joint diseases	70 (5.8)
	Endocrine disorder diseases	128 (10.6)
	Total	1215 (100)
Previous/home medications by group	Gastrointestinal medicines	112 (8.7)
	Cardiovascular medicines	371 (28.9)
	Respiratory medicines	198 (15.5)
	Anti-infective medicines	330 (25.7)
	Central nervous system medicines	22 (1.7)
	Medicines used in musculoskeletal and joint disease	61 (4.7)
	Vitamins, minerals, and herbal supplements	71 (5.5)
	Medicines used in endocrine disorders and contraceptives	81 (6.3)
	Others^b^	39 (3.0)
	Total	1285 (100)
Source of medication fee	Health insurance	223 (35.1)
	Out-of-pocket payment	412 (64.9)
Management of preadmission medications	Autonomous	366 (57.6)
	Assistance from family	212 (33.4)
	Assistance from health workers	57 (9.0)
Medication adherence	High	104 (16.4)
	Medium	135 (21.3)
	Low	396 (62.4)

^a^ Total: some patients had more than one comorbidities; *N* frequency, *%* Percent, *FHCSH* Felege Hiwot comprehensive specialized hospital, *TGCSH* Tibebe Ghion comprehensive specialized hospital, ^**b**^ Others: obstetrics and gynecological medications, blood products and medicines affecting the blood, antineoplastic and supportive medicines, dermatological agents, eye/ear medicine

### The source of information for MedRec and the magnitude of medication discrepancies

**Fig. 2:**
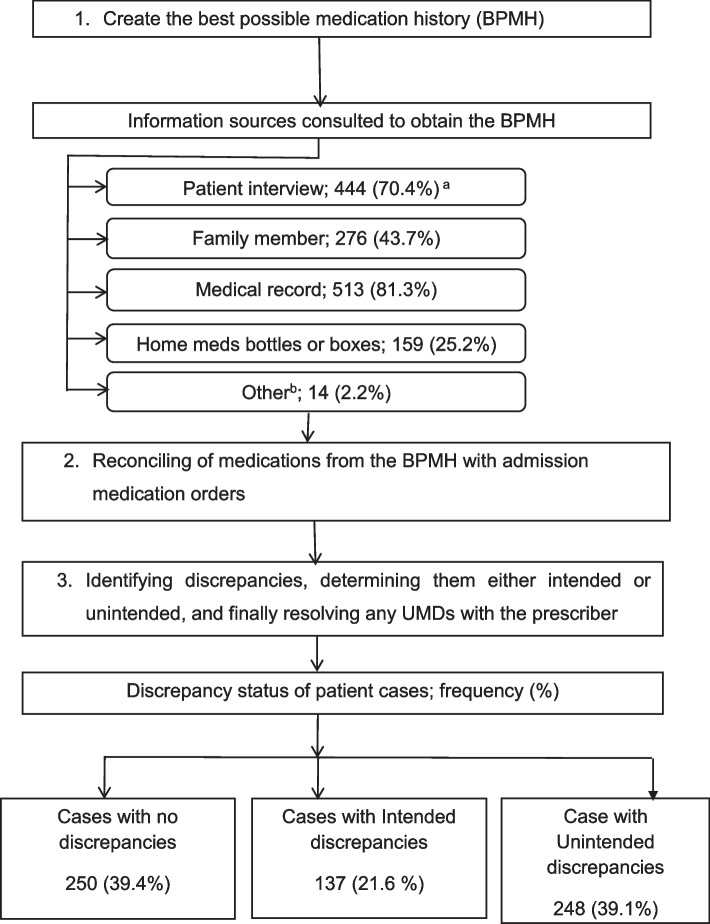
MedRec process and the magnitude of medication discrepancies. ^a^Cumulative percentage exceeds 100% since more than one source was used for a patient; ^b^Other: communication with prescribing physician, prescriptions from other health institutions, previous discharge summaries, community pharmacy; BPMH: best possible medication history; UMDs: unintended medication discrepancies; FHCSH: Felege Hiwot comprehensive specialized hospital; TGCSH: Tibebe Ghion comprehensive specialized hospital

At least two sources of information for BPMH were checked for all patients, two sources for 343 (54.0%) and more than two sources for 292 (46.0%) patients. Patient interviews (70.4%), medical records (81.3%), and family member interviews (43.7%) were the top three sources of information. The majority (60.6%) of participants had at least one medication discrepancy (either intentional or unintentional). At least one UMD was found in more than one-third (39.1%) (95% CI = 35.2, 43.0) of patients (Fig. 2).

### The class and magnitude of medications involved in UMDs, and the types of UMDs

Of 1285 home medications, about 388 (30.2%) medications were involved in UMDs. The most frequent type of UMD was omission (41.75%), followed by wrong dose (21.9%) of medication. According to the ATC classification, cardiovascular medicines (32.5%) (e.g. hydrochlorothiazide, furosemide, amlodipine, digoxin, enalapril, warfarin, propranolol, atorvastatin, and aspirin), the drugs of the alimentary tract and metabolism (22.4%) (e.g. metformin, Insulin, glibenclamide, propylthiouracil (PTU), vitamin B_12,_ folic acid), and anti-infective medicines for systemic use (18.8%) (e.g. Co-trimoxazole, TB/HIV medications, amoxicillin-clavulanic acid, ceftriaxone) accounted the highest proportions of UMDs (Supplementary file 1).

### Proximal causes leading to the UMDs & pharmacists’ interventions

The main proximal causes of UMDs were the patient’s lack of knowledge (60.1%) and the physician’s lack of awareness of the medications (17.3%). From a total of 442 interventions recommended by the pharmacists, the majority (75.3%) were fully accepted. The most common types of interventions were the addition of medication (65.9%) and dose adjustment (35.4%). Most of the interventions not accepted were related to the omission of vitamins, minerals, contraceptives, and dermatological agents, which were assessed by the physicians as unnecessary during hospitalization (Table 3).

**Table 3:** Proximal causes leading to UMDs and pharmaceutical intervention with its rate of acceptance

The proximal causes leading to UMD	Frequency (%)
physician’s lack of awareness	43 (17.3)
Patient’s lack of knowledge	149 (60.1)
Dosage form confusion	2 (0.8)
Brand and generic name similarity	3 (1.2)
Unknown	51 (20.6)
Total	248 (100.0)
Type of pharmaceutical intervention	Frequency (%)^a^
Adjust dose	87 (35.4)
Adjust route	43 (17.5)
Adjust frequency	17 (6.9)
Add medication	162 (65.9)
Discontinue medication	44 (17.9)
Highlight drug-drug interactions	15 (6.1)
Highlight drug-disease interactions	6 (2.4)
Educate the patient	68 (27.7)
The acceptance rate of pharmaceutical interventions	Frequency (%)
Fully accepted	333 (75.3)
Partially accepted	37 (8.4)
Not accepted	72 (16.3)
Total	442 (100)

^a^ Cumulative percentage exceeds 100% since more than one intervention was used for a patient, *FHCSH* Felege Hiwot comprehensive specialized hospital, *TGCSH* Tibebe Ghion comprehensive specialized hospital, % Percent, *UMD* Unintended medication discrepancy

### Associated factors of unintended medication discrepancies

After controlling for the effects of potentially confounding variables, patients with polypharmacy at admission were 5.47 times more likely to have UMD as compared to patients without polypharmacy at admission (adjusted odds ratio (AOR) = 5.47; 95% CI = 3.52, 8.48; *p*-value < 0.001). Similarly, for a unit increase in the number of information sources consulted, the revealing of UMD increases by a factor of 2.83 (AOR = 2.83; 95% C.I = 1.98, 4.05; *p*-value < 0.001). Patients who had age ≥ 65 have 2.13 times the odds of having UMD as compared to patients who were below 65 years old (AOR = 2.13; 95% CI = 1.40, 3.24; *p*-value = 0.001). For a unit increase in the number of comorbidities, the occurrence of UMD also increases by a factor of 1.29 (AOR = 1.29, 95% CI = 1.07, 1.56; *p*-value = 0.008). As compared to patients who have high level of medication adherence, patients with medium and low adherence were 2.93 (AOR = 2.93; 95% CI = 1.19, 7.16; *p*-value = 0.019) and 11.13 times (AOR = 11.13; 95% CI = 5.17, 23.98; *p*-value < 0.001) more likely to have UMD, respectively (Table 4).

**Table 4:** Factors associated with the UMD identified through MedRec

Variable	Category	UMD		COR (95% CI)	AOR (95% CI)	*P*-Value
		No	Yes			
Age (years)	< 65	267	130	1	1	
	≥ 65	120	118	2.02 (1.45, 2.81)	2.13 (1.40, 3.24)	0.001
Medication adherence	High adherence	94	10	1	1	
	Medium Adherence	109	26	2.24 (1.03, 4.89)	2.93 (1.19, 7.16)	0.019
	Low adherence	184	212	10.83 (5.48, 21.40)	11.13 (5.17, 23.98)	< 0.001
Number of previous/home medications	< 5	373	224	1	1	
	≥ 5	14	24	2.86 (1.45, 5.63)	1.76 (0.75, 4.16)	0.271
Source of medication fee	Free	126	97	1	1	
	Payment	261	151	0.75 (0.54, 1.05)	0.76 (0.491, 1.17)	0.210
Number of medications at admission	< 5	230	44	1	1	
	≥ 5	157	204	6.79 (4.63, 9.97)	5.47 (3.52, 8.48)	< 0.001
Type of admission	Emergency	177	83	1	1	
	Direct /scheduled	210	165	1.68 (1.20, 2.33)	1.16 (0.75, 1.79)	0.517
Number of information sources consulted to obtain BPMH^a^		387	248	3.52 (2.60, 4.76)	2.83 (1.98, 4.05)	< 0.001
Number of admissions in the last year		387	248	1.33 (1.12, 1.58)	1.18 (0.951, 1.47)	0.131
Number of comorbidities		387	248	1.19 (1.03, 1.38)	1.29 (1.07, 1.56)	0.008

^a^ *BPMH* Best possible medication history, *COR* Crude odds ratio, *AOR* Adjusted odds ratio, 95% *CI* 95 percent confidence interval, *UMD* Unintended medication discrepancy, *FHCSH* Felege Hiwot comprehensive specialized hospital, *TGCSH* Tibebe Ghion comprehensive specialized hospital

## Discussion

This study aimed at assessing the magnitude and associated factors of UMDs identified through medication reconciliation during patient admission to the internal medicine wards of Felege Hiwot and Tibebe Ghion comprehensive specialized hospitals in Bahir Dar city, Northwest Ethiopia.

More than one-third (39.1%) (95% CI = 35.2, 43.0) of patients had at least one UMD in their medication regimen, and 388 (30.2%) medications were involved in the UMDs. This finding is consistent with the results reported from studies done in St. Paul’s hospital millennium medical college, Ethiopia (41.4%) [49]; Chicago, Illinois (35.9%) [21]; Saint-Antoine Hospital, Paris, France (42%) [24], and Dhahran, Saudi Arabia (37%) [19]. However, it is higher than the studies conducted at a university hospital in Harar, Eastern Ethiopia (32.2%) [28]; Eastern provinces of Saudi Arabia (23%) [17]; Paris, France (18.8%) [25]; Strasbourg, France (33.2%) [29], and a university hospital in Brazil (20%) [38]. On the contrary, the figure in our study is lower than the studies done in Beirut, Lebanon (46.1%) [27]; Spanish hospitals (49.5%) [18]; Jeddah, Saudi Arabia (48.3%) [26], and Kenyatta National Hospital, Kenya (63.2%) [48]. The difference may be attributed to a slight variation in the study population, point of care transition, study area, a slight variation in the definitions of medication discrepancies, and level of care in developing and developed countries. For example, the study in Kenya was done on elderly diabetes patients. The study at a university hospital in Harar, Eastern Ethiopia was done upon internal transition. This study affirms that the point of patient admission to the internal medicine wards is one of the most key vulnerable areas for the occurrence of UMD. Adding to the existing evidence, this study emphasizes the importance of the implementation of MedRec upon patient care transition in hospitals in low-income settings.

In this study, omission (41.75%) and wrong dose (21.9%) of medications were the most frequently identified UMDs. Similarly, many of the previous studies reported that omission (35.49–98.3%) followed by wrong dose (7- 40.3%) of medications were the most frequent UMDs [12, 15–31]. This may be because most patients and their attendants do not have the habit and awareness of the importance of proactively informing their treating physicians regarding their home medicines. Consequently, they may discontinue or receive the wrong dose of their home medicine without the awareness of the treating physicians upon hospital admission. So that taking a BPMH more than one source of information and performing a structured formal MedRec process may be crucial to prevent treatment interruptions due to omission and to avert the wrong dose of medications upon patient admission [4, 9].

According to the WHO’s ATC code, medications for the cardiovascular system (32.5%), alimentary tract and metabolism (22.42%), and anti-infective for systemic use (18.8%) were the main categories of medications involved in UMDs. This finding is supported by previous studies [22, 24]. Therefore, during medication reconciliation giving more attention to patients taking these groups of medications can significantly contribute to improving patient safety.

In this study, patients' lack of knowledge (60.1%) was the most common cause of UMDs. This finding is supported by many previous studies [27, 38, 63]. Understanding proximal causes leading to medication discrepancies may help to orient the MedRec process and can direct where the emphasis should be targeted to avoid potential UMDs [64]. In addition, patient education regarding their pharmacotherapy could have a great contribution to reducing UMDs.

In this study, the pharmaceutical interventions involved patients, caregivers, and treating physicians. Patients and caregivers were counseled on the proper use of medication, and they were taught the correct application of eye/ear drops and/or ointments, accordingly. All 388 medications involved in UMDs required intervention by the prescribing physician. Some of UMDs needed more than one intervention, as in what was reported in many of the previous studies [23, 27, 38]. From 422 pharmacist interventions, most (78.90%) of them were accepted and resulted in a modification in the patient’s care. Studies done in Brazil (71%) [38], Lebanon (64.6%) [27], and Belgium (72.3%) also reported similar acceptance rates [23]. However, in a study conducted at Cooper University, the acceptance rate was relatively low (22%) [44]. The high acceptance rate of the interventions is a good indicator that an implementation of a pharmacists-led MedRec would improve the communication and teamwork between health care professionals to identify and rectify UMDs. This indicates how significant the role of pharmacists could be in the detection and prevention of medication discrepancies and drug-related problems (DRPs) in Ethiopia as these problems are highly prevalent (70%) in Ethiopia [65].

Patients with polypharmacy at admission were 5.47 times more likely to have UMD as compared to patients without polypharmacy (AOR = 5.47; 95% CI = 3.52, 8.48). This finding is consistently reported in many other previous studies [19, 20, 37, 42, 66, 67]. This may be because as the number of medications that a patient takes increases, it becomes more difficult for the treating physicians to keep an accurate medication history for each medicine in the routine process considering their workload. Thus, separate, formally delegated, and responsible professionals for performing BPMH and MedRec would have a great benefit for a patient with polypharmacy.

For a unit increase in the number of comorbidities, the occurrence of UMD increases by a factor of 1.29 (AOR = 1.29; 95% CI = 1.07, 1.56). Previous studies also reported the presence of a significant association between the number of comorbidities and UMD [18, 29, 38]. This may be because healthcare providers most likely focus on the medications of the primary or working diagnosis than home medications, and patients and their caregivers may fail to bring home medications used for comorbid conditions. So, patients with comorbidity should be the other important target for a formal MedRec process.

For a unit increase in the number of information sources consulted, the revealing of UMD increases by a factor of 2.83 (AOR = 2.83; 95% C.I = 1.98, 4.05). This is supported by the findings from many other previous studies [18, 19, 38]. WHO recommended that at least two sources of information should be used to determine the accuracy of a patient's medication history [4, 39], and should be implemented for every patient.

Patients who had age ≥ 65 have 2.13 times the odds of having UMD as compared to patients who were below 65 years old (AOR = 2.13; 95% CI = 1.40, 3.24). This finding is supported by many of the previous studies [16, 17, 21, 37, 42, 43, 66, 68]. This may be because, in addition to having multiple disease states requiring treatment with multiple medications, older patients may have less knowledge of their medication, which is the most common reason for UMD identified in the current study. The implementation of MedRec by prioritizing more risky groups like older patients would have paramount importance to improve patient safety.

As compared to highly adherent patients, medium and low adherent patients were 2.93 (AOR = 2.93; 95% CI = 1.19, 7.16) and 11.13 (AOR = 11.13; 95% CI = 5.17, 23.98) times more likely to be associated with UMD, respectively. This is important evidence to the existing literature. Because non-adherence can be cognitive (intentional) or behavioral (unintentional), it may greatly influence the medication history. For example, patients may not have the motivation or willingness to tell the treating physician the truth about intentional discontinuation and change in the dose of their home medication. The patient may forget to take their home medicines as prescribed or the dosing regimen is too complicated for the patient’s abilities. As a result, they may give incorrect information to the treating physician unintentionally, which intern results in UMD [69]. This implies that MedRec, which recommends the use of more than one source of information BPMH, provides a more accurate history in non-adherent patients. So that good medication history should encompass adherence to medication in addition to all currently and recently prescribed drugs, previous adverse drug reactions including hypersensitivity reactions, and any over-the-counter medications including herbal or alternative medicines [70].

### The strength and limitations of the study

Being the first study conducted on UMDs upon patient admission in Ethiopia, the authors tried to put their best effort to show the magnitude, types, mostly involved classes of medicines, proximal causes, associated factors of UMDs, and the acceptance rate of the interventions made by pharmacists. However, this study is not without any limitations. Not including all transitions of care like internal transfer and discharge, pediatric, surgical, and gynecology-obstetrics patients may be the limitation of this study. In addition, the potential severity of the UMDs was not rated by using indexes like the National Coordinating Council for Medication Error Reporting and Prevention (NCCMERP). NCCMERP is a Medication Error Index that classifies an error according to the severity of the outcome. It requires the expert panel, which might be external to the internal medicine ward and with different expertise and responsibilities related to pharmacotherapy.

## Conclusions

This study concluded that UMDs were considerably high upon hospital admission to the internal medicine wards. Omission and the wrong dose of medication were common. Medicines for cardiovascular disease, alimentary tract and metabolism, and anti-infective for systemic use were mostly involved. Patient's lack of knowledge of their medication regimen was the most frequent proximal cause of UMDs. Most of the interventions recommended by pharmacists were acceptable. Patients age ≥ 65, polypharmacy at admission, low and medium adherence, an increased number of comorbidity, and an increase in the number of information sources checked for the BPMH had a significant association with UMDs. Implementing a formal MedRec to reduce UMDs at the transition of care is highly recommended. The hospitals could assign and integrate pharmacists with a healthcare team to perform a formal MedRec at the transition of care to improve patient safety by giving priority to high-risk individuals. Further studies are recommended to assess pharmacist-led MedRec across all transition points of care in both pediatric and adult patients.

## Supplementary Information

**Additional file 1.** UMDs by discrepancy type and WHO ATC codes.

## Data Availability

The datasets generated during and analyzed during the current study are not publicly available due to a large amount of text material but are available from the corresponding author on reasonable request.

## Abbreviations

ACDS: Adherence in Chronic Diseases Scale
ADE: Adverse Drug Event
AOR: Adjusted Odds Ratio
BPMH: Best Possible Medication History
COR: Crude Odds Ratio
FHCSH: Felege Hiwot Comprehensive Specialized Hospital
IQR: Interquartile range
ISMP: Institute of Safe Medication Practices
MedRec: Medication Reconciliation
NCCMERP: National Coordinating Council for Medication Error Reporting and Prevention
OTC: Over the Counter
SOP: Standard Operating Protocol
SPSS: Statistical Package for the Social Sciences
TGCSH: Tibebe Ghion Comprehensive Specialized Hospital
UMD: Unintended Medication Discrepancy
WHO: World Health Organization
