# Supplementary material for: Unintended medication discrepancies and associated factors upon patient admission to the internal medicine wards: identified through medication reconciliation
Source: BMC Health Serv Res. 2022 Oct 15;22:1251. doi: 10.1186/s12913-022-08628-5 (PMC9571466; doi:10.1186/s12913-022-08628-5)
Supplement: Supplementary file 1 — Additional file 1. UMDs by discrepancy type and WHO ATC codes. [file 12913_2022_8628_MOESM1_ESM.docx]

Supplementary file 1: UMDs by discrepancy type and WHO ATC codes

| UMDs  by type  Frequency (%) | Medication group by WHO ATC codes; frequency (%) | | | | | | | | | | | | | | |
| --- | --- | --- | --- | --- | --- | --- | --- | --- | --- | --- | --- | --- | --- | --- | --- |
|  | A | B | C | D | G | H | J | L | M | N | P | R | S | V |  |
| Omission  162 (41.75) | 36 (41.4) | 2  (1.0) | 78  (61.9) | 1  (16.7) | 3  (0.6) | 3  (0.4) | 18  (24.7) | 0  (0) | 4  (0.4) | 5  (29.4) | 2  (28.6) | 6  (18.8) | 3  (0.5) | 1  (14.3) |  |
| Commission 47 (12) | 6  (7) | 0  (0) | 13  (10.3) | 0  (0) | 1  (0.2) | 0  (0.0) | 11  (15) | 1  (0.5) | 1  (0.1) | 4  (23.5) | 2  (28.6) | 5  (15.6) | 1  (16.7) | 2  (28.6) |  |
| Wrong dose  85 (21.9) | 21  (24) | 0  (0) | 17  (13.5) | 3  (0.5) | 0  (0) | 0  (0) | 22  (30) | 0  (0) | 3  (0.3) | 2  (11.8) | 1  (14.3) | 12  (37.5) | 2  (33.3) | 2  (28.6) |  |
| Wrong route  45 (12) | 13  (15) | 0  (0) | 10  (7.9) | 1  (16.7) | 0  (0) | 1  (0.13) | 12  (16.4) | 0  (0) | 1  (0.1) | 2  (11.8) | 1  (14.3) | 4  (12.5) | 0  (0) | 0  (0) |  |
| Wrong frequency  21 (5.4) | 5  (5.7) | 0  (0) | 3  (2.4) | 1  (16.7) | 1  (0.2) | 2  (25) | 4  (5.5) | 1  (0.5) | 0  (0) | 1  (5.9) | 0  (0) | 3  (9.4) | 0  (0) | 0  (0) |  |
| Wrong duration  6 (1.5) | 0  (0) | 0  (0) | 2  (1.6) | 0  (0) | 0  (0) | 0  (0) | 2  (2.7) | 0  (0) | 1  (0.1) | 1  (5.9) | 0  (0) | 0  (0) | 0  (0) | 0  (0) |  |
| Duplication  12 (3.0) | 2  (2.3) | 0  (0) | 2  (1.6) | 0  (0) | 0  (0) | 1  (0.1) | 3  (4) | 0  (0) | 0  (0) | 1  (5.9) | 1  (14.3) | 1  (3.1) | 0  (0) | 1  (14.3) |  |
| Drug interaction  10 (2.6) | 4  (4.6) | 0  (0) | 1  (0.8) | 0  (0) | 0  (0) | 1  (0.1) | 1  (1.4) | 0  (0) | 0  (0) | 1  (5.9) | 0  (0) | 1  (3.1) | 0  (0) | 1  (14.3) |  |
| Total  388 (100) | 87 (22.4) | 2 (0.5) | 126 (32.5) | 6 (1.6) | 5 (1.3) | 8 (2.0) | 73 (18.8) | 2 (0.5) | 10 (2.6) | 17 (4.4) | 7 (1.8) | 32 (8.2) | 6 (1.6) | 7 (1.8) |  |

WHO ATC codes: A: alimentary tract and metabolism; B: blood and blood-forming organs; C: cardiovascular system; D: dermatological; G: genitourinary system and sex hormones; H: Systemic hormonal preparations, excluding sex hormones and Insulin; J: Anti-infective for systemic use; L: Antineoplastic and immune-modulating Agents; M: muscular-skeletal system; N: the nervous system; P: antiparasitic products, insecticides and repellents; R: respiratory system; S: sensory organs; V: various; UMDs: Unintended medication discrepancies; WHO: World Health Organization, ATC: Anatomic Therapeutic Chemical classification; FHCSH: Felege Hiwot comprehensive specialized hospital; TGCSH: Tibebe Ghion comprehensive specialized hospital; N: frequency; %: percent
